# Efficacy of Biologically Active Food Supplements for People with Atherosclerotic Vascular Changes

**DOI:** 10.3390/molecules27154812

**Published:** 2022-07-27

**Authors:** Natalia Pleshkova, Boisjoni Tokhiriyon, Andrei Vekovtsev, Valeriy Mikhailovich Poznyakovsky, Valentina Lapina, Madina Atlaevna Takaeva, Vladimir Nikolaevich Sorokopudov, Elena Valeryevna Karanina

**Affiliations:** 1 Department of Economics and Management, Kemerovo State University, 650000 Kemerovo, Russia; 9530076903@mail.ru; 2 Department of Management, Entrepreneurship and Engineering, Ural State University of Economics, 620144 Ekaterinburg, Russia; b.tohiriyon@usue.ru; 3 Department of Production and Science, Scientific Production Association Artlife, 634034 Tomsk, Russia; pvm1947@bk.ru; 4 Scientific and Educational Center for Applied Biotechnology and Nutrition, Kemerovo State Medical University, 650029 Kemerovo, Russia; boisjoni@mail.ru; 5 Scientific and Educational Center for Agricultural Raw Materials Processing and Food Technology, Kuzbass State Agricultural Academy, 650056 Kemerovo, Russia; 6 Department of Biology and Chemistry, Kadyrov Chechen State University, 364024 Grozny, Russia; 6950990456@mail.ru; 7 All-Russian Scientific Research Institute of Medical and Aromatic Plants, 117216 Moscow, Russia; bt-881311@mail.ru; 8 Department of Economics and Finance, Vyatka State University, 610000 Kirov, Russia; 9920285200@mail.ru

**Keywords:** BAFS, functional foods, peripheral atherosclerosis, combination therapy, efficacy

## Abstract

The current paper deals with the development of a new biologically active food supplement (BAFS) aimed at treating atherosclerosis. Since atherosclerosis is considered to be a disease of aging, the composition of the supplement includes such essential minerals as magnesium and potassium, which are commonly used to prevent atherosclerosis, as well as vitamins C, E and the B-group vitamins in order to address the needs of the elderly. The authors outline the supplement-manufacturing technology and discuss the clinical trial undertaken by patients, aged about 60 years, with peripheral atherosclerosis. The research methodology focuses on studying the effectiveness of the developed supplement by assessing the influence of the active ingredients on treating metabolic disorders. To establish the efficacy of the supplement, blood tests, ultrasound and physical examinations were applied. The combination therapy resulted in improved metabolism and an overall better performance of the cardiovascular system; therefore, the BASF can be recommended as part of combination therapy to prevent and treat atherosclerotic and age-related changes in blood vessels.

## 1. Introduction

Recent technological advances allow supplement producers to offer different finished products to cater for the needs of children and adults. In Russia, for instance, a popular range of ‘Pantoshka’ dragees helps to provide children with the recommended daily intake of essential vitamins and minerals, while ‘Vitaminnii Balsam’ (a thick vitamin drink), composed of antler products and/or herbs, assists adults in boosting metabolism and preventing infections in cold seasons. Taking into account the expeditiously aging population, there is a growing interest in supplements for senior citizens. As a rule, the elderly tend to consume fewer nutrients and, thus, experience a lack of essential minerals and vitamins. Moreover, due to gradual cellular damage, there are several common age-related diseases that need to be addressed. One of the common conditions that can develop with age is atherosclerosis [1,2].

Diseases caused by atherosclerosis are still the main cause of mortality among the able-bodied population in most countries of the world [1,2,3,4,5,6,7,8].

The effect of atherosclerosis on the occurrence and development of cardiovascular pathologies is immense. First, atherosclerosis increases the susceptibility of blood vessels to spasms. Seemingly, this happens because debris of the vascular wall and plasma impregnation can irritate the sensitive ends of vasoconstrictors embedded in the vessel walls, which are much more sensitive to irritation than vasodilators. Therefore, even weak spasms can cause a strong vasopressor effect, which enhances and accelerates angina attacks and hypertensive crises [3,4]. Second, atherosclerotic plaques, as well as thickening of the vascular wall, narrow the lumen of blood vessels and disrupt the flow of blood to organs, including the heart muscle and brain tissue. Atherosclerotic plaques can completely close the lumen of the feeding artery and cause corresponding organ failure. Third, the atherosclerotic process damages the vascular endothelium and causes blood clots, since one of the most important factors of thrombus formation is the intima integrity violation. Blood clots can result in various degrees of blood supply disruption. They can also break off and turn into emboli [5]. Finally, at the stage of an atheromatous ulcer, debris, entering the bloodstream, also turns into an embolus, which can be carried by the blood and can clog small blood vessels. This process is especially dangerous in case of an ulcerated atheromatous plaque, when it is localized in the lumen of the functionally terminal coronary and cerebral vessels. Debris flows through the bloodstream into smaller branches of these vessels, clogs them and can cause the development of myocardial infarction or ischemic stroke [3,4,5].

Despite significant advances in cardiology and clinical pharmacology, the issue of effective and safe treatment of patients with circulatory failure induced by atherosclerosis is still topical. In addition to pharmaceutical-drug development, it has become necessary to create new anti-atherosclerotic medicines of natural origin, including biologically active food supplements with a multicomponent composition, which can act at various stages of the disease pathogenesis. This approach will promote diet therapy as part of combination therapy and improve preventive measures for people who do not show a clinical manifestation of the disease [9,10,11,12,13,14,15].

Latterly, a number of scientists have explored the impact of proper nutrition on atherosclerosis, analyzing n-3 fatty acids, vitamins and herbs and looking for the right composition, best route and suitable doses [16,17,18]. Acknowledging the significance of further research, we aimed to develop a new dietary supplement composed of a mix of minerals and vitamins and designed to meet the needs of senior citizens. The supplement intake is part of combination therapy for people with peripheral atherosclerosis.

## 2. Materials and Methods

### 2.1. The Research Design

To evaluate the developed supplement, we conducted a randomised study. The sample size of 20 participants was calculated to be the most cost- and time-effective to conduct the research. Since the focus of our study was the critical issue of treating atherosclerosis, to meet the selection criteria, the participants were to be male, older than 55, diagnosed with atherosclerosis and having risk factors for the development of cardiovascular diseases.

The study was conducted in compliance with the guidelines of the Declaration of Helsink, the principles of ICH GCP, ethical requirements set out in the Directive of the European Union 2001/20/EC, as well as the requirements of the Russian legislation. Each patient signed an informed consent form to participate in the study.

According to the research protocol, 20 men with peripheral atherosclerosis (the main group) were under observation. The average age of the men was 61.3 ± 4.3 years. The control group consisted of 15 male volunteers with a similar diagnosis and an average age of 63.2 ± 3.5 years. All volunteers had a risk factor for adverse development of cardiovascular disease (CVD) as they smoked between 1/2 and 1 pack of cigarettes a day. The main group of patients took a tablet of the food supplement 3 times a day for a month, while the control group patients did not.

Before, during and after the course of treatment, we analyzed the severity (the extent of the disease) of peripheral atherosclerosis, the nutritional status, the coagulation system and the blood lipid spectrum. Clinical, laboratory, instrumental (ultrasound examination) and biostatistical research methods were used in the study, which was performed on the basis of the General Surgery Department of the Siberian State Medical University under the supervision of Professor V.I. Tikhonov, the Doctor of Medical Science.

### 2.2. Product-Manufacturing Technology

The manufacturing technology of the encapsulated food supplement was developed. It has the following stages: raw-material preparing; mixture for encapsulation preparing; raw-material sieving and blending; encapsulating and sorting capsules by their appearance; and packing, packaging and labeling [12,13,14,15,19,20,21,22,23,24].

The order of dosing the product ingredients was determined. Control at this production stage is carried out according to conformity of the raw-material name, quantity and series to the technology chart. It should be mentioned that some ingredients need to undergo preparation before mixing. Coenzyme Q10, calcium pantothenate, pyridoxine hydrochloride and thiamine mononitrate are mixed together with 2 kg of lactose and then sieved, while chromium picolinate, folic acid and cyanocobalamin are, first, mixed with lactose and, then, thoroughly pressed with a pounder. After pressing, 1.5 kg of lactose is added.

The composition ingredients are sieved through a vibrosieve with a 1 mm hole diameter. There cannot be lumps or foreign inclusions. The ingredient screening is reground with a hammer mill and sieved again.

Blending is performed in a V-shaped mixer, where 100 kg of blend is mixed for 1 h. When pressed with a pounder, the blend must be free of lumps and foreign inclusions.

The obtained blend goes to the encapsulating stage. The average weight is checked every 30 minutes by weighing 20 capsules. The average weight and individual capsule weight should not exceed 5%. Every 20 min, capsules are also checked for appearance. Any damaged capsules are discarded. Then, capsules are dedusted, weighed and passed to the packing and packaging stage. Three packs are sent to the quality control laboratory and another three packs are sent to the arbitration collection of medicine samples. The product is stored according to the technical documentation.

Qualitative and quantitative product composition as well as the recommended daily intake can be found in Table 1.

The supplement functional properties and nutritional value, as well as quality and safety indicators, were determined following thorough organoleptic, physico-chemical, sanitary–hygienic and toxicological tests (Table 2). The assessment of the nutritional value of the dietary supplement was performed using high-performance liquid chromatography and atomic absorption spectroscopy [25].

## 3. Results and Discussion

Patients with peripheral atherosclerosis who took the food supplement demonstrated positive dynamics of their clinical condition, although there was no significant difference with the control group.

The main primary symptom of peripheral obliterating atherosclerosis (POA) is known to be leg pain when walking, which gradually disappears after the person stops [3,4]. This typical symptom is observed in most cases and defined as “intermittent claudication”. It is the result of tissue ischemia that occurs during exercise due to restriction of blood flow, while with rest, sufficient blood supply resumes. The data in Table 3 demonstrate the tendency towards decreasing the peripheral atherosclerosis severity.

Upon the supplement intake, 6 patients out of 20 (30% of cases) recorded that intermittent claudication occurred after they walked about 500 meters compared to 100 m before the treatment. The control group patients did not show this result.

The biochemical status of patients under observation was studied (Table 4).

The combination of traditional conservative therapy of peripheral atherosclerosis and the food supplement intake had a positive effect on the body functioning. We could observe the tendency for glucose lowering and transaminase activity reduction.

Atherosclerosis is a chronic disease. Its pathogenesis significantly depends on the anti-inflammatory body response [9]. In this regard, it is worth noting that patients who took the food supplement demonstrated a C-reactive protein (CRP) decrease up to its total elimination in the blood serum. The control group patients did not show this effect (*p* < 0.01).

Blood coagulation analysis did not reveal significant changes caused by the product intake but blood rheological properties improved (Table 5).

Improved blood rheological properties manifested in the tendency for lower total fibrinogen level in the serum of the patients who took the specialized product.

The product intake as part of combination therapy demonstrated its ability to normalize cholesterol metabolism (Table 6). At the end of treatment, the main-group patients demonstrated VLDL level reduction by 1.3 times (*p* < 0.05), when compared with the control group patients. At the beginning of treatment, it was 1.4.

We can see an atherogenic-coefficient decrease due to an HDL-cholesterol increase.

These indicators of the main-group patients differed from the control group patients by 1.1 and 1.2 times, respectively. The study also revealed a significant decrease in triglycerides level in the blood (15%) in the main-group patients. In addition, the level of triglycerides in the serum of the main-group patients was 21% lower than that before the combination therapy (Table 6).

Cholesterol metabolism normalization also affected the arteries’ state. Six volunteers who took the supplement agreed to the second ultrasound examination of lower-limb arteries after treatment. We observed the tendency for loose-plaque compaction in three patients. The blood flow increased by 12%. One patient showed calcification.

The level of homocysteine in the blood of the main-group patients dropped from 16.4 ± 2.5 µmol/L at the beginning of treatment to 15.9 ± 2.1 µmol/L at its end. This drop contributed positively to the disease dynamics. The control group patients showed a drop from 16.1 ± 2.5 µmol/L to 16.0 ± 2.3 µmol/L.

Since the product’s active substances improve the state of blood vessel walls, microcirculation was evaluated by pressing the nail bed (Table 7).

We revealed a significant increase in blood flow velocity under the influence of the food supplement intake, which indicates the capillary-strengthening effect of the nutrition factor and its beneficial effect on the microcirculation.

According to blood chemistry and ECG results, there was no significant difference in the disease dynamics before and after treatment with the food supplement. This fact demonstrates that the product does not influence homeostasis indicators. The volunteers also showed good product acceptability. The study of blood coagulation system indicators did not reveal any significant deviations during the treatment either. Moreover, we observed a favorable tendency for blood rheological properties’ improvement and cholesterol metabolism normalization in patients with peripheral atherosclerosis who took the specialized food supplement. The VLDL-cholesterol level decrease indicates that healthy people can take the specialized product to prevent atherosclerotic lesions of the arteries. A slight change in homocysteine level at the end of the supplement administration may indicate that the administration period should be longer. The product intake improved microcirculation. The tone of the capillary bed in patients with peripheral atherosclerosis who took the product as part of combination therapy improved. The membrane-stabilizing activity of the product antioxidants was demonstrated as the inflammatory-process severity decreased. Taking into account the significance of age-related changes in the development of vascular atherosclerosis and well-known theoretical principles of its development, we can say that the product intake slows down the disease development. A high antioxidant potential of the product plays a certain part in the disease progression slowdown as antioxidants can reduce oxidative stress and the severity of nicotine abuse effects. They can also provide the body with microelements (potassium and magnesium), which prevent an excess fluid build-up in the body and have their own antihypertensive potential.

The combination therapy resulted in normalizing the condition of patients with peripheral atherosclerosis. They recorded milder clinical symptoms, less pain while walking and better metabolism indicators shown by blood biochemistry tests.

**Figure 1:**
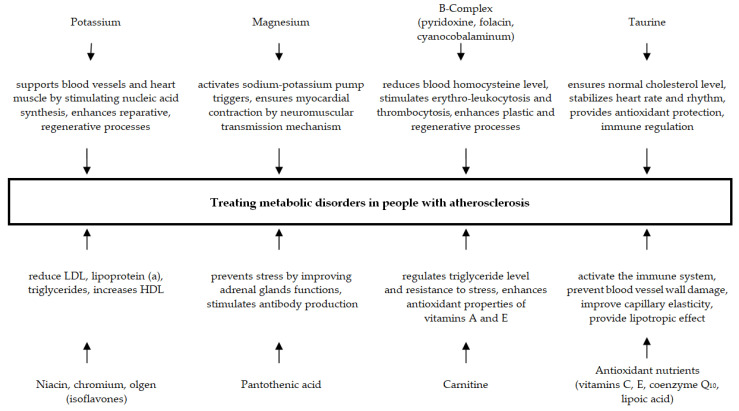
Mechanism of the food supplement influence on improving metabolism disorders in people with peripheral atherosclerosis.

Both the obtained results and available medical literature data allow us to look into the possible mechanism of the diet therapy influence on people with vascular system disorders (Figure 1) [26,27,28,29,30,31,32,33,34].

It seems appropriate to outline the most significant aspects of atherosclerosis pathogenesis in order to understand the effective ways of treating the disease, which may include the nutrition factor.

Peripheral atherosclerosis is one of the most common types of lesions in the arterial bed. It is quite often observed in patients with atherosclerosis of the coronary and carotid arteries, although it is often asymptomatic. Hyperhomocysteinemia is currently considered as an independent, modifiable CVD risk factor. An increase in blood homocysteine level by 5 μmol/L increases the risk of atherosclerotic vascular damage by 80% in women and 60% in men [35,36].

High homocystein level causes atherosclerosis development and progression due to several reasons. First, homocystein in blood plasma is easily oxidized. During sulfhydryl group oxidation, reactive substances of oxygen are formed, which have a toxic effect on vascular endothelial cells. Homocystein leads to the inhibition of nitric oxide effects, reduces its bioavailability and affects tissue’s sensitivity to it [37]. Second, under homocysteinemia, prostacyclin synthesis decreases and arterial smooth-muscle-cell growth increases. Homocystein promotes protein disulfide derivatives’ formation, LDL and VLDL build-up in the cell membranes and intercellular space and lipoprotein oxidation [38]. It also leads to a decrease in the sulfur-containing glycosaminoglycan synthesis, which causes reduced blood vessel elasticity. Oxidized lipids stimulate the expression of pro-inflammatory cytokines, directly inactivate nitric acid oxide and are toxic to endotheliocyte. They result in reduced vessel elasticity and dilatation, which, to a larger extent, is due to endothelial dysfunction. Third, the imbalance of cholesterol metabolism and the neurogenic factor contribute to the atherosclerotic process progression.

In addition, hypertension can also cause atherosclerosis. This is a separate disease, when a persistent high blood pressure is the main, and sometimes the only, symptom. Hypertension is caused by a primary violation of vascular tone as a result of neurohumoral regulation disorder [3,4,5].

## 4. Conclusions

As with the majority of studies, our trial has certain limitations that could be addressed in future research. First, the participants selected for the trial were all male. Second, the risk factor for all the participants was smoking. Therefore, these limitations should be taken into account when interpreting the results of our study.

Considering all the obtained results, we can conclude that the nutrition factor, i.e., the developed and tested biologically active food supplement intake, helps improve the functional state of the vascular system and blood microcirculation, and reduces the severity of cholesterol metabolism disorders. Since particular nutrients become crucial for general well-being, the needs of senior citizens have to be specifically catered for. We recommend taking the product as part of combination therapy for people with atherosclerosis as well as for healthy people to prevent atherosclerotic and age-related changes in blood vessels.

## Figures and Tables

**Table 1:** The chemical composition.

Ingredients	Content, mg (1 Capsule Weight—750 mg)	The Recommended Daily Intake, mg
Magnesium oxide Magnesium	133 75	420
Potassium orotate Potassium	100 20	3500
Potassium chloride Potassium	80 42	3500
Taurine	80	1250
Magnesium lactate Magnesium	48.8 5	420
Soy isoflavones extract “Solgen” Soy isoflavones	25 10	50
L-Carnitine	15	1000
Vitamin C (ascorbic acid)	14	100
Lipoic acid	6.0	30
Vitamin B3 (nicotinic acid)	4.0	20
Coenzyme Q10	3.0	200
Vitamin E (tocopherol acetate)	2.0	15
Vitamin B5 (calcium pantothenate)	1.0	5
Vitamin B6 (pyrodixin hydrochloride)	0.4	2
Vitamin B1 (thiamine mononitrate)	0.3	1.5
Chromium picolinate Chromium	0.08 0.01	0.4
Vitamin B9 (folic acid)	0.04	0.4
Vitamin B12 (cyanocobalamin)	0.0006	0.3

**Table 2:** The product nutritional value indicators.

Indicators	Content, mg (1 Capsule Weight—750 mg)
Vitamin E	2.0 (1.8–2.3)
Vitamin B1	0.3 (0.27–0.35)
Vitamin B3	4.0 (3.6–4.6)
Vitamin B5	1.0 (0.9–1.15)
Vitamin B6	0.4 (0.36–0.46)
Vitamin B9	0.04 (0.03–0.05)
Vitamin B12, mcg	0.6 (0.54–0.7)
Vitamin C, minimum	14.0
Lipoic acid	6.0 (5.4–7.0)
Coenzyme Q10	3.0 (2.7–3.5)
L-Carnitine	15 (13.5–17.3)
Taurine	80 (72–92)
Magnesium	80 (72–92)
Potassium	185 (157.5–212.8)
Chromium, minimum	0.01
Soy isoflavones, minimum	10.0

**Table 3:** Patient distribution according to peripheral atherosclerosis severity during treatment.

Severity II III IV	Main Group (n = 20)		Control Group (n = 15)		Significance Level	
	Before Treatment	After Treatment	Before Treatment	After Treatment	Before Treatment	After Treatment
I (minor) II (moderate) III (major) IV (extreme)	4	7	5	6	0.31	0.52
	11	10	6	6	0.07	0.13
	5	3	4	3	0.60	0.51
	0	0	0	0	-	-

**Table 4:** Biochemical status of patients with peripheral atherosclerosis during treatment.

Indicators	Main Group (n = 20)		Control Group (n = 15)		Significance Level	
	Before Treatment	After Treatment	Before Treatment	After Treatment	Before Treatment	After Treatment
Protein, g/L	72.63 ± 2.81	71.85 ± 1.95	72.34 ± 4.25	73.11 ± 13.21	0.54	0.52
Glucose, µmol/L	5.21 ± 0.23	4.79 ± 0.75	5.16 ± 0.79	5.11 ± 0.94	0.34	0.06
AST, u/L	35.53 ± 2.18	31.42 ± 1.56	36.60 ± 2.93	35.12 ± 2.14	0.64	0.37
ALT, u/L	29.33 ± 2.54	26.49 ± 1.58	28.25 ± 1.91	28.24 ± 2.07	0.63	0.22
Bilirubin total, µmol/L	10.61 ± 1.65	10.50 ± 1.69	10.95 ± 2.05	10.41 ± 1.54	0.54	0.56
Thymol test, u.	1.92 ± 0.32	1.58 ± 0.41	2.06 ± 0.38	2.00 ± 0.59	0.45	0.29
CRP	4.00 ± 0.50	2.7 ± 0.37	4.5 ± 0.48	3.9 ± 0.41	0.65	0.31

**Table 5:** Blood coagulation properties of patients with peripheral atherosclerosis during treatment.

Indicators	Main Group (n = 20)		Control Group (n = 15)		Significance Level	
	Before Treatment	After Treatment	Before Treatment	After Treatment	Before Treatment	After Treatment
Total fibrinogen, g/L	3.9 ± 0.85	3.00 ± 0.64	3.22 ± 0.64	3.16 ± 0.85	0.55	0.19
INR, u	0.88 ± 0.09	0.80 ± 0.06	0.89 ± 0.04	0.87 ± 0.05	0.54	0.36
SFMC, g/L	3.56 ± 1.04	3.34 ± 0.68	3.54 ± 1.09	3.46 ± 1.05	0.51	0.22

**Table 6:** Blood lipid spectrum indicators of people with chronic venous insufficiency (CVI) during treatment.

Indicators	Main Group (n = 20)		Control Group (n = 15)		Significance Level	
	Before Treatment	After Treatment	Before Treatment	After Treatment	Before Treatment	After Treatment
Total cholesterol	8.2 ± 1.5	7.5 ± 1.2 *	7.9 ± 1.3	7.7 ± 1.4	0.25	0.27
VLDL-cholesterol	2.53 ± 0.41	1.75 ± 0.21 *	2.56 ± 0.28	2.34 ± 0.31	0.22	0.036
LDL-cholesterol	3.16 ± 0.66	3.02 ± 0.58 *	3.8 ± 0.78	3.14 ± 0.69	0.45	0.08
HDL-cholesterol	1.11 ± 0.10	1.31 ± 0.08 *	1.14 ± 0.22	1.19 ± 0.16	0.46	0.044
Triglycerides	2.42 ± 0.44	1.88 ± 0.35 *	2.39 ± 0.48	2.20 ± 0.49	0.35	0.047
Atherogenic coefficient **	7.39 ± 1.09	5.72 ± 0.84 *	6.92 ± 0.75	6.47 ± 0.52	0.18	0.045
Homocysteine, μmol/L	16.4 ± 2.5	15.9 ± 2.1	16.1 ± 2.9	16.0 ± 2.3	0.41	0.43

Note: * *p* < 0.05 when indicators are compared before and after treatment in a group (Student′s *t*-test was applied to obtain the statistical significance.).** Atherogenic Coefficient (AC) = (TC-HDL-c)/HDL-c.

**Table 7:** Nail bed response to pressing in patients with CVI and peripheral atherosclerosis during treatment.

Groups	Before Treatment	After Treatment	Significance Level
Main group, n = 15	2.0 ± 0.4	1.5 ± 0.2 *	0.041
Control group, n =10	2.1 ± 0.3	1.9 ± 0.4 *	0.18

Note: * *p* < 0.05 (W-Wilcoxon test).

## Data Availability

The data presented in this study are available on request from the corresponding authors.

## Sample Availability

Samples of the compounds are not available from the authors.
